# Supplementary material for: Molecular co-assembled strategy tuning protein conformation for cartilage regeneration
Source: Nat Commun. 2024 Feb 19;15:1488. doi: 10.1038/s41467-024-45703-3 (PMC10876949; doi:10.1038/s41467-024-45703-3)
Supplement: Supplementary file 1 — Supplementary Information [file 41467_2024_45703_MOESM1_ESM.pdf]

# Supplementary Information

# Molecular co-assembled strategy tuning protein conformation for cartilage regeneration

Chengkun Zhao[1,2#], Xing Li[1,2#], Xiaowen Han[3], Zhulian Li[1,2], Shaoquan Bian[4], Weinan Zeng[5], Mingming Ding[6], Jie Liang[1,2,7], Qing Jiang[1,2], Zongke Zhou[5], Yujiang Fan[1,2], Xingdong Zhang[1,2], Yong Sun[1,2*]

[1] National Engineering Research Center for Biomaterials, Sichuan University, 29[#] Wangjiang Road, Chengdu, Sichuan, 610064, P. R. China.

[2] College of Biomedical Engineering, Sichuan University, 29[#] Wangjiang Road, Chengdu, Sichuan 610064, P. R. China.

[3] NHC Key Laboratory of Nuclear Technology Medical Transformation, Mianyang Central Hospital, Mianyang, Sichuan, 621099, P. R. China

[4] Shenzhen Institutes of Advanced Technology, Chinese Academy of Sciences, Shenzhen 518055, P. R. China.

[5] Department of Orthopedic Surgery and Orthopedic Research Institution, West China Hospital, Sichuan University, Chengdu 610041, China

[6] College of Polymer Science and Engineering, State Key Laboratory of Polymer Materials Engineering, Sichuan University, Chengdu, 610065, P. R. China

[7] Sichuan Testing Center for Biomaterials and Medical Devices, Sichuan University, 29# Wangjiang Road, Chengdu 610064, P. R. China.

***Corresponding author**.

Email: sunyong8702@scu.edu.cn (Y.S.)

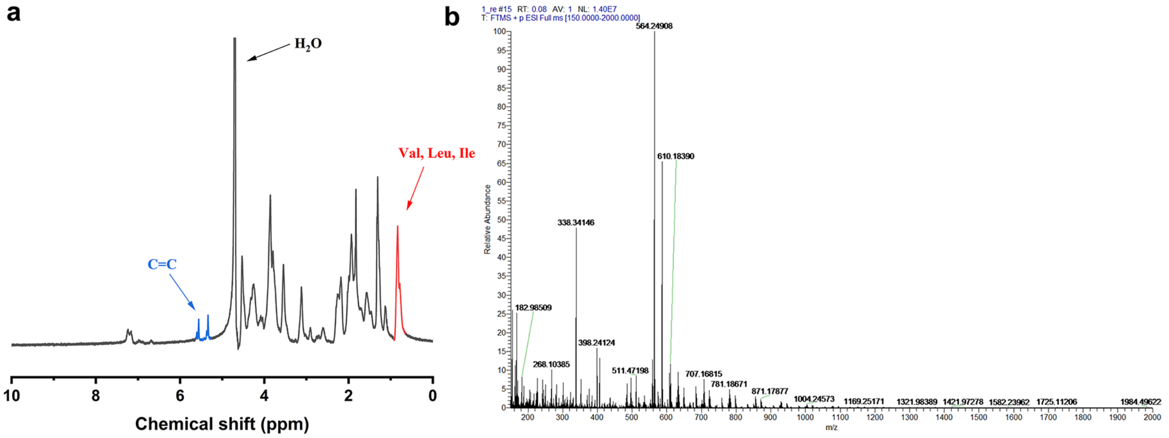


**Supplementary Figure 1. Characterization of GelMA and BPAA-GFF. (a)** The $^{1}$H NMR spectrum of GelMA, new resonant peaks at 5.35 and 5.6 ppm in $^{1}$H-NMR spectra confirmed the existence of C=C groups. GelMA with 76.74% substitution degree was successfully prepared[1]. **(b)** Electrospray ionization mass spectrometry for BPAA-GFF. The molecular weight *m/z* of BPAA-GFF synthesized by ESI-MS data is 564.25, which confirmed the successful synthesis of BPAA-GFF.

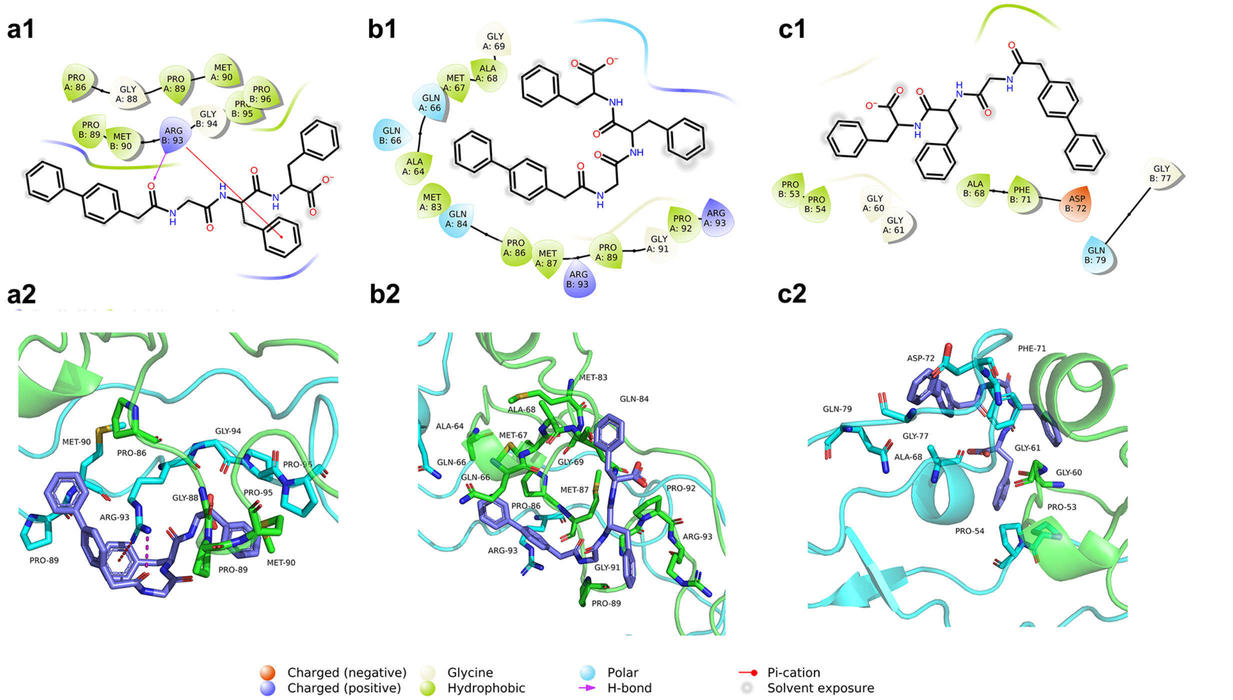


**Supplementary Figure 2. 2D and 3D pictures of representative interaction sites between BPAA-GFF and GelMA in molecular docking analysis. (a1, b1, c1)** 2D view and **(a2, b2, c2)** 3D view of the interaction between BPAA-GFF and GelMA. AutoDock software was employed to conduct molecular docking. The best binding site was predicted using SiteMap software.

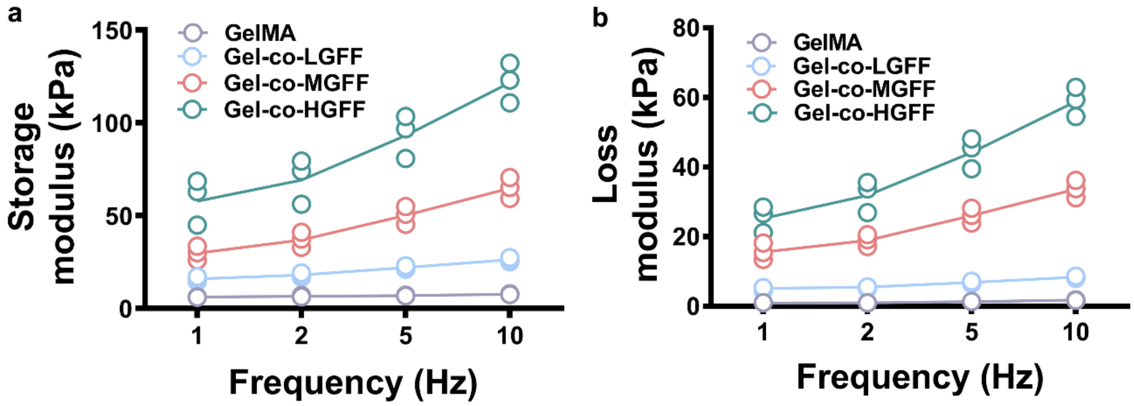


**Supplementary Figure 3. The mechanical properties of hydrogels. (a)** Storage modulus and **(b)** loss modulus in different frequencies. The storage modulus (G′) and loss modulus (G″) of hydrogels ($n = 3$ independent samples) were determined by a dynamic mechanical analyzer (TA-Q800, USA) instrument with the frequency from 1 to 10 Hz at constant room temperature. Data are mean ± s.d.

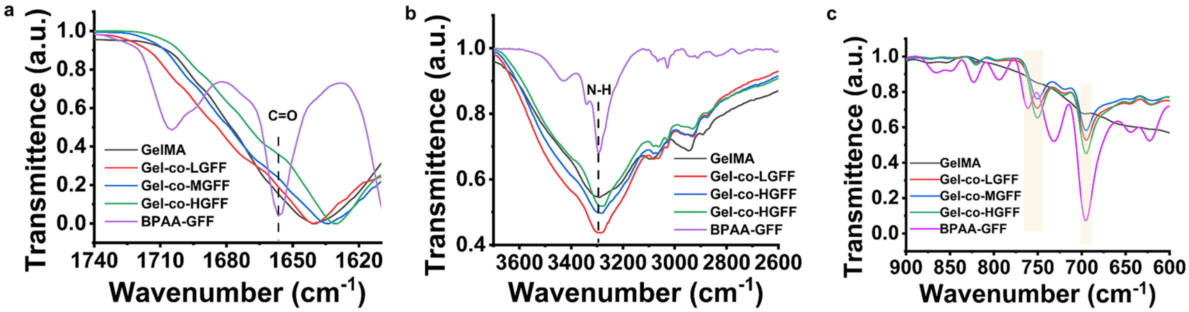


**Supplementary Figure 4. The evaluation of H-bonds and Pi-Pi stacking interactions.** The Fourier Infrared characteristic absorption peak of **(a)** C=O stretching vibration, **(b)** N-H stretching vibration, and **(c)** C=H bending vibration in biphenyl. Fourier transform infrared spectra indicating H-bonding and Pi-Pi interactions. The slight red shift for characteristic peaks of C=O and N-H stretching vibrations suggested H-bond enhancement in co-assembled hydrogels. The red shift of C=H characteristic peak in biphenyl also revealed Pi-Pi stacking interactions. These spectra were detected by Fourier transform infrared spectroscopy (Nicolet 6700, USA) with a 2 $cm^{-1}$ resolution and presented in the wavenumber range of 600-900 $cm^{-1}$, 1620-1740 $cm^{-1}$, and 2600-3700 $cm^{-1}$.

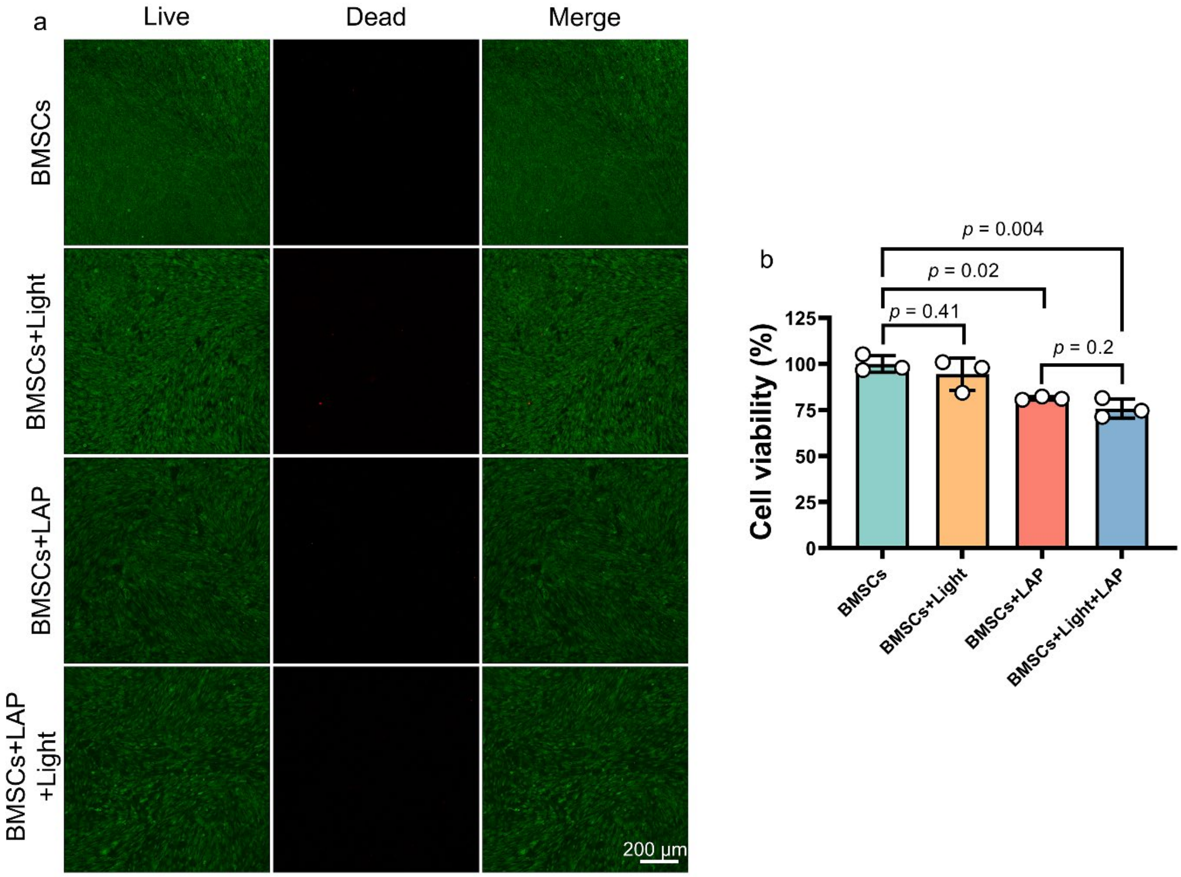


**Supplementary Figure 5. The effect of photocuring on BMSCs. (a)** Live/dead images of BMSCs under different conditions. The four groups were BMSCs (without treatment), BMSCs + light (UV irradiation), BMSCs + LAP (0.5% LAP in medium, *w/v*), and BMSCs + LAP + Light (0.5% LAP in medium and initiated by UV light, *w/v*). **(b)** CCK-8 analysis for BMSCs in four groups. Data are mean ± s.d. Statistical analyses were performed with one-way analysis of variance (ANOVA), followed by Tukey's multiple comparison post hoc test, *$p < 0.05$, **$p < 0.01$, and ***$p < 0.001$.

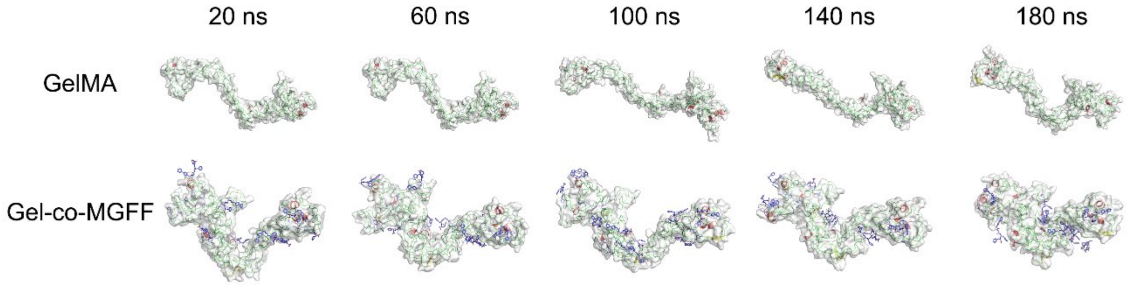


**Supplementary Figure 6. The structure oscillation for GelMA and Gel-co-MGFF during molecular dynamics simulation.** Molecular dynamics simulations were performed using the Gromacs 5.1.5 open-source software package. The simulated system was set in a confined environment with the temperature set to 300 K, pH set to 7.4, and the pressure to one atmosphere 1 bar.

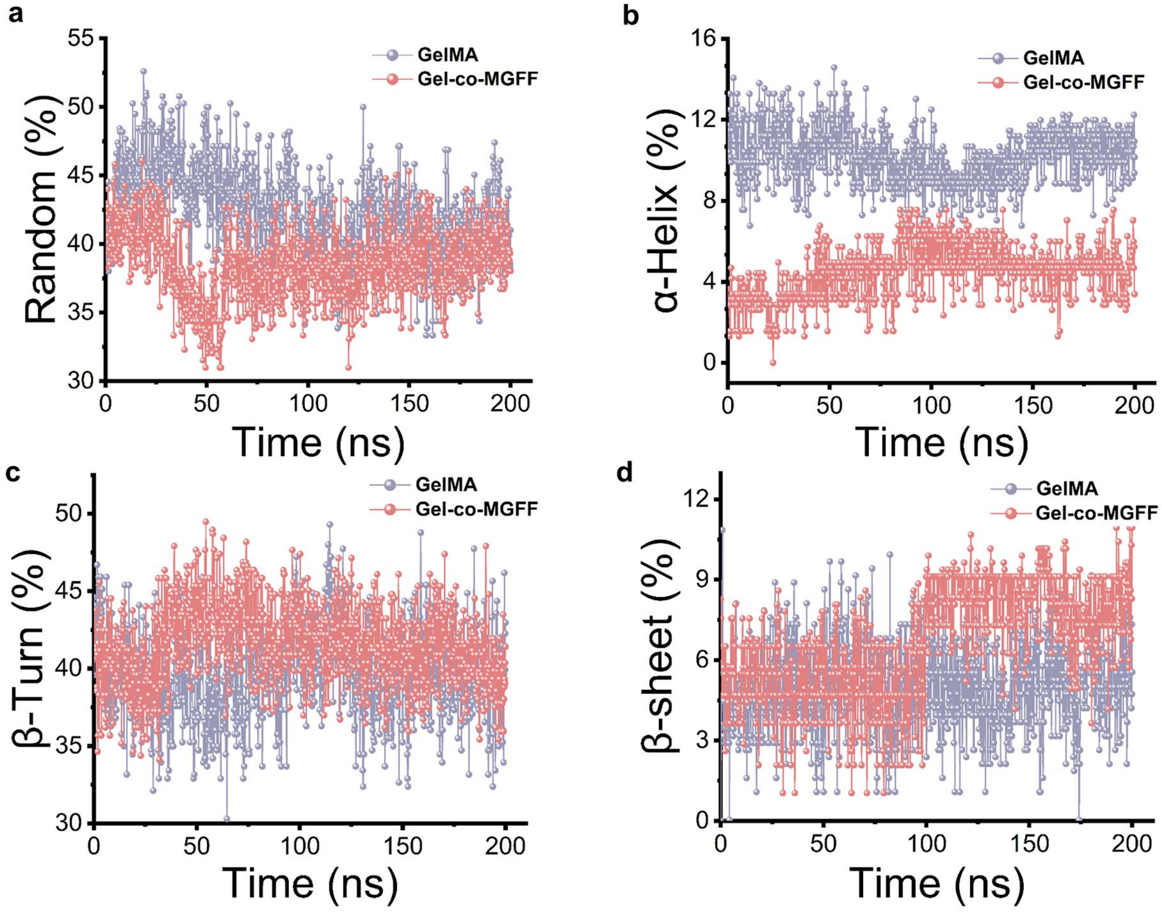


**Supplementary Figure 7. The oscillation of secondary structure content calculated from molecular dynamics simulation. (a)** Random structure content. **(b)** α-Helix content. **(c)** β-turn content. **(d)** β-sheet content. Molecular dynamics simulations were performed using the Gromacs 5.1.5 open-source software package. This figure exhibited the increase of β-sheet content in Gel-co-MGFF during the simulation time (0-200 ns).

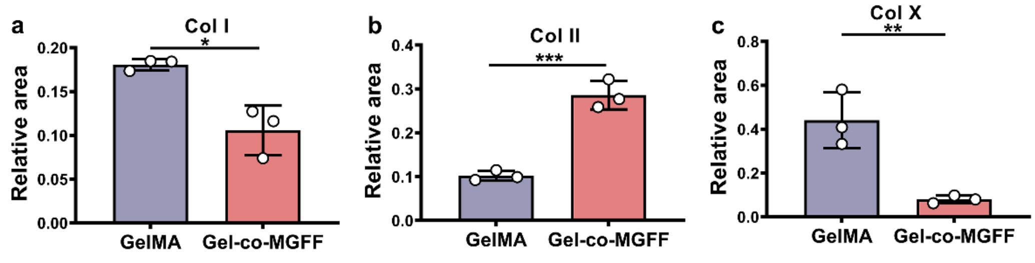


**Supplementary Figure 8. Semi-quantitative analysis for matrix *in vivo*.** The semi-quantitative analysis from CLSM images of **(a)** Col I, **(b)** Col II, and **(c)** Col X staining after BMSCs-laden hydrogels transplanted into nude mice subcutaneously (normalized to nucleus staining area) (**p* = 0.01, ****p* < 0.001, ***p* = 0.008) *n* = 3 cells examined three independent experiments. Data are mean ± s.d. Statistical analyses were performed with Student's unpaired t-test, **p < 0.05, **p < 0.01, and ***p < 0.001.*

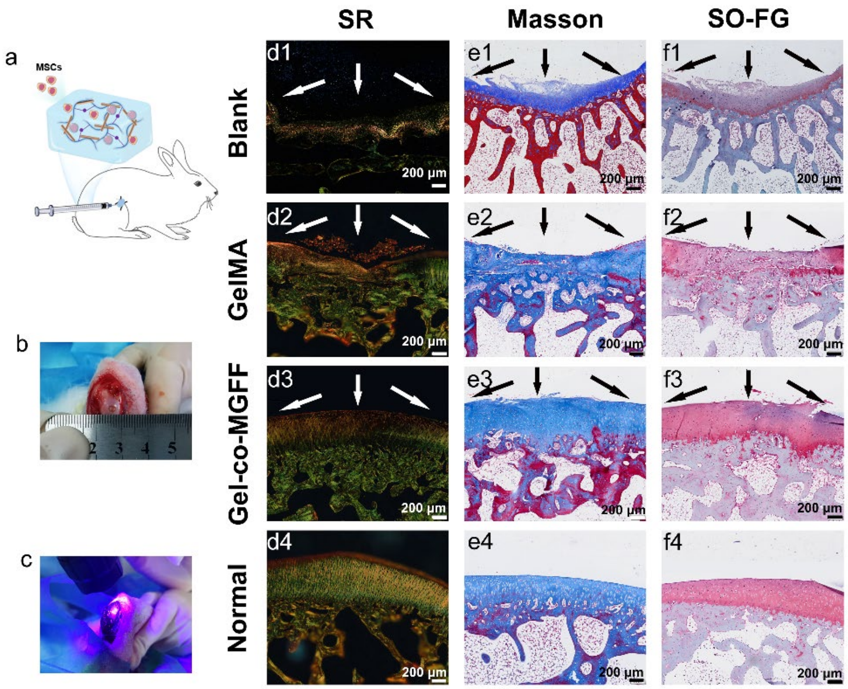


**Supplementary Figure 9. The schematic diagram and histological staining of the *in vivo* experiment. (a)** Schematic diagram of BMSCs-laden hydrogels injected into the rabbit knee defective area (Φ = 3 mm). **(b, c)** The process of injection *in situ* and light irradiation. **(d1-f4)** Representative pictures of Sirius-red, Masson's trichrome, and Safranin O-Fast green staining after 3 months of implantation. The arrows point to the defective area.

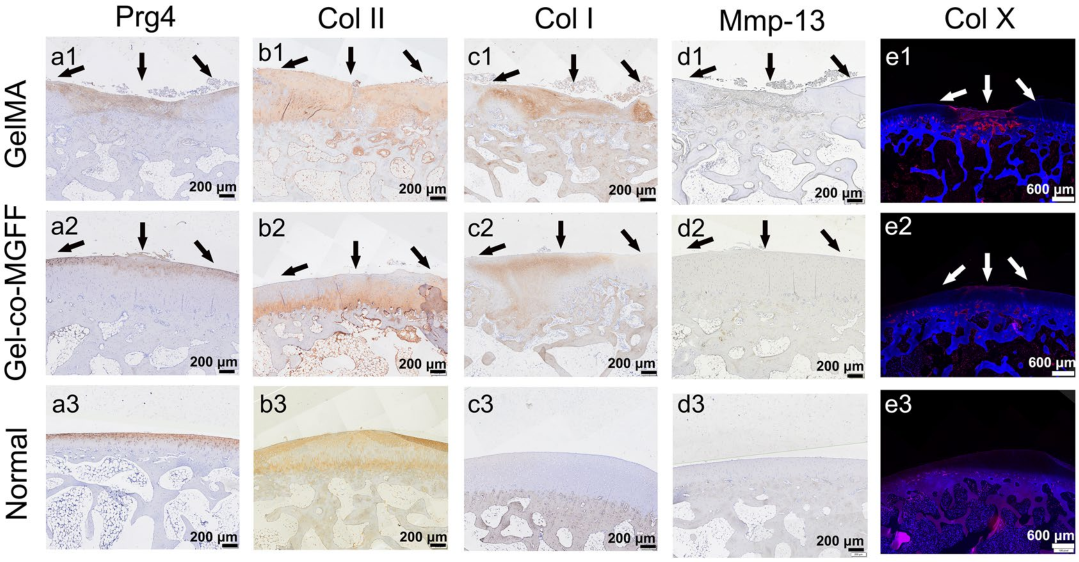


**Supplementary Figure 10. The immunohistochemical and immunofluorescence staining of regenerated tissue after three months. (a1-d3)** Immunohistochemistry staining for Prg 4, Col II, Col I, and Mmp-13, and **(e1-e3)** Immunofluorescence staining for Col X. The arrows point to the defective area.

**Supplementary Table 1. Peak position and content for all types of secondary structure**

| | GelMA | | Gel-co-MGFF | |
|---|---|---|---|---|
| | Peak position (cm$^{-1}$) | Content (%) | Peak position (cm$^{-1}$) | Content (%) |
| β-sheet | 1619 | 50.5 | 1612 | 58.5 |
| | 1631 | | 1623 | |
| | 1641 | | 1633 | |
| | 1691 | | 1641 | |
| | 1702 | | 1695 | |
| β-turn | 1672 | 17.7 | 1670 | 16.5 |
| | 1681 | | 1681 | |
| α-helix | 1664 | 14.3 | 1658 | 11.5 |
| random | 1652 | 17.5 | 1648 | 13.5 |

**Supplementary Table 2. Synthesis sequence of qRT-PCR primers.**

| **Primer** | **Sequence (5′-3′)** |
|---|---|
| *Gapdh*-F | TCGGAGTGAACGGATTTGGC |
| *Gapdh*-R | TTCCCGTTCTCAGCCTTGAC |
| *Col I*-F | GTCGATGGCTGCACGAAAAA |
| *Col I*-R | GGGCCAACGTCCACATAGAA |
| *Col II*-F | TGATAAGGATGTGTGGAAGCCG |
| *Col II*-R | CAGGCAGTCCTTGGTGTCTTC |
| *Col X*-F | TCCCAGAACCCAGAATCCATC |
| *Col X*-R | GGTTGTGGGCCTTTTATGCC |
| *Agg*-F | GGCCACTGTTACCGTCACTT |
| *Agg*-R | GTCCTGAGCGTTGTTGTTGAC |
| *Mmp-13*-F | AGCTTCCCAACCGCATTGAT |
| *Mmp-13*-R | GGTCTTGCCCGTGTCTTCAA |

## References

1. Ovsianikov, A. et al. Laser fabrication of three-dimensional cad scaffolds from photosensitive gelatin for applications in tissue engineering. *Biomacromolecules* **12**, 851-858 (2011).
