# Supplementary material for: Molecular co-assembled strategy tuning protein conformation for cartilage regeneration
Source: Nat Commun. 2024 Feb 19;15:1488. doi: 10.1038/s41467-024-45703-3 (PMC10876949; doi:10.1038/s41467-024-45703-3)
Supplement: Supplementary file 3 — Reporting Summary [file 41467_2024_45703_MOESM3_ESM.pdf]

Corresponding author(s): Yong Sun

Last updated by author(s): Jan 2, 2024

Reporting Summary

Nature Portfolio wishes to improve the reproducibility of the work that we publish. This form provides structure for consistency and transparency in reporting. For further information on Nature Portfolio policies, see our Editorial Policies and the Editorial Policy Checklist.

Statistics

For all statistical analyses, confirm that the following items are present in the figure legend, table legend, main text, or Methods section.

n/a	Confirmed	
☐	☒	The exact sample size (*n*) for each experimental group/condition, given as a discrete number and unit of measurement
☐	☒	A statement on whether measurements were taken from distinct samples or whether the same sample was measured repeatedly
☐	☒	The statistical test(s) used AND whether they are one- or two-sided<br>*Only common tests should be described solely by name; describe more complex techniques in the Methods section.*
☐	☒	A description of all covariates tested
☐	☒	A description of any assumptions or corrections, such as tests of normality and adjustment for multiple comparisons
☐	☒	A full description of the statistical parameters including central tendency (e.g. means) or other basic estimates (e.g. regression coefficient) AND variation (e.g. standard deviation) or associated estimates of uncertainty (e.g. confidence intervals)
☐	☒	For null hypothesis testing, the test statistic (e.g. *F*, *t*, *r*) with confidence intervals, effect sizes, degrees of freedom and *P* value noted<br>*Give P values as exact values whenever suitable.*
☒	☐	For Bayesian analysis, information on the choice of priors and Markov chain Monte Carlo settings
☐	☒	For hierarchical and complex designs, identification of the appropriate level for tests and full reporting of outcomes
☐	☒	Estimates of effect sizes (e.g. Cohen's *d*, Pearson's *r*), indicating how they were calculated

Our web collection on statistics for biologists contains articles on many of the points above.

Software and code

Policy information about availability of computer code

Data collection	MestReNova software(Version 10.0.1), Confocal laser scanning microscope (LSM 880, Zeiss), CFX96 real-time PCR detection system (Bio-Rad CFX Manager 3.0), TEM (TECNAI G2 F20 S-TWIN at 200 kV), FT-IR spectroscopy (Nicolet 6700), SEM (HITACHI S-800), DSC (METTLER TOLEDO), Electromechanical universal testing machine(Shimadzu Autograph AGS-X), Dynamic mechanical analyzer (DMA, TA-Q800), UV spectrometer (Lambda 850, PerkinElmer), Gromacs package(Version 5.1.5), Alphafold2, Avogadro software(Version 1.2.0). All softwares used in this study have been described in detail in supplementary methods.
Data analysis	Commercial Fiji ImageJ 1.8.0, OMNIC(Version 8.2.0.387), Origin 8.0, Graphpad Prism 8.4, Case Viewer 2.1 software (Pannoramic MIDI, 3D HISTECH, Hungary), and ZEN software (Carl Zeiss Microscopy GmbH, Version 2.3.69.1000)

For manuscripts utilizing custom algorithms or software that are central to the research but not yet described in published literature, software must be made available to editors and reviewers. We strongly encourage code deposition in a community repository (e.g. GitHub). See the Nature Portfolio guidelines for submitting code & software for further information.

Data

Policy information about availability of data

All manuscripts must include a data availability statement. This statement should provide the following information, where applicable:

- Accession codes, unique identifiers, or web links for publicly available datasets
- A description of any restrictions on data availability
- For clinical datasets or third party data, please ensure that the statement adheres to our policy

All data needed to evaluate the conclusions in the paper are present in the paper and/or the Supplementary Materials. Additional data related to this paper may be requested from the authors.

Research involving human participants, their data, or biological material

Policy information about studies with human participants or human data. See also policy information about sex, gender (identity/presentation), and sexual orientation and race, ethnicity and racism.

Reporting on sex and gender	NA
Reporting on race, ethnicity, or other socially relevant groupings	NA
Population characteristics	NA
Recruitment	NA
Ethics oversight	NA

Note that full information on the approval of the study protocol must also be provided in the manuscript.

Field-specific reporting

Please select the one below that is the best fit for your research. If you are not sure, read the appropriate sections before making your selection.

[x] Life sciences [ ] Behavioural & social sciences [ ] Ecological, evolutionary & environmental sciences

For a reference copy of the document with all sections, see nature.com/documents/nr-reporting-summary-flat.pdf

Life sciences study design

All studies must disclose on these points even when the disclosure is negative.

Sample size	The sample sizes were estimated based on previous studies (G.G. Lu et al., Nat. commun., 13, 2499, 2022 and X. Li et al., Adv. Funct. Mater. 33, 2212738, 2023) and have been listed in the manuscript. For all in vitro studies, at least three random samples were selected for statistical analysis in each experiment based on the effect size and overlap between distributions. For all in vivo studies, at least three random samples were applied for statistical analysis in each experiment.
Data exclusions	No data points were excluded from analysis.
Replication	All experimental findings were reliably reproduced, and attempts at replication of experimental findings were successful. All, material preparation, processing and characterization were performed with a minimum of three samples. Both in vitro and in vivo experiments were replicated at least three times to confirm experimental trends prior to publication.
Randomization	Different treatments were assigned to animals randomly divided into control and experimental groups. After three months, three random independent samples in each group were used for immunohistochemistry, immunofluorescence, and histological staining. For histological, immunohistochemistry, and immunofluorescence staining, the field of view was randomly selected for analysis. For q-PCR, samples were randomized and analyzed with standard approaches. Cell culture samples were assigned randomly, with control and experimental groups analysed in identical conditions to minimise potential covariates.
Blinding	For in vitro studies, blinding of test samples was not applicable since differences between groups were clear to the researchers by naked eye. However, the data were anonymized for statistical analysis. For in vivo studies, different researchers were randomly involved in animal group distribution, operation, and evaluation. All investigators were blinded to group allocation during data collection. All assessments of functional recovery (regenerated cartilage, ICRS scores, etc.) were performed in a blinded manner. Immunostaining expression level was also performed blinded and the data were anonymized for statistical analysis.

Reporting for specific materials, systems and methods

We require information from authors about some types of materials, experimental systems and methods used in many studies. Here, indicate whether each material, system or method listed is relevant to your study. If you are not sure if a list item applies to your research, read the appropriate section before selecting a response.

Materials & experimental systems

n/a	Involved in the study
☐	☒ Antibodies
☐	☒ Eukaryotic cell lines
☒	☐ Palaeontology and archaeology
☐	☒ Animals and other organisms
☒	☐ Clinical data
☒	☐ Dual use research of concern
☒	☐ Plants

Methods

n/a	Involved in the study
☒	☐ ChIP-seq
☒	☐ Flow cytometry
☒	☐ MRI-based neuroimaging

Antibodies

Antibodies used

anti-Col I, Rabbit, NOVUS, NB600-408, 1:200 (IF, IHC)
anti-Col II, Mouse, NOVUS, NB600-844, 1:500 (IF, IHC)
anti-Col X, Mouse, Invitrogen, MA5-14268, 1:200 (IF)
anti-Sox9, Rabbit, Bioss, bs-10725R, 1:200 (IF)
anti-Mmp-13, Rabbit, Invitrogen, PA5-33940, 1:100 (IHC)
anti-Prg4, Mouse, Sigma-Aldrich,MABT401,1:500 (IHC)
anti-CD86, Rabbit, Affinity, DF6332, 1:400 (IF)
anti-CD206, Rabbit, Affinity, DF4149, 1:400 (IF)
anti-CD19, Rabbit, NOVUS, NBP2-15782, 1:500 (IF)
anti-IgG, Rabbit, Bioss, bs-0296R-Cy7, 1:500 (IF)
Cy3 conjugated Goat Anti-Mouse IgG (H+L), Servicebio, GB21301, 1:200 (IF)
Cy3 conjugated Goat Anti-Rabbit IgG (H+L), Servicebio, GB21303, 1:200 (IF)
FITC conjugated Goat Anti-Rabbit IgG (H+L),Servicebio, GB22303, 1:200 (IF)
FITC conjugated Goat Anti-Mouse IgG (H+L),Servicebio, GB22301, 1:200 (IF)

Validation

Positive and negative controls of specific-binding (for each of the florescent labeled targets) were included in each experiment.
Specific criteria are used to determine antibody eligibility for each application by the manufacturer. We typically test each antibody in multiple repeated test for the same application. Antibody specificity is verified by a continuous gene knockout (KO)-validation program by the manufacturer. To ensure that different batches of the same antibody produced the same results, the manufacturer also assessed the differences between batches using conformance tests. Antibodies were used at the dilutions recommended by the manufacturer and the statements can be found on the manufactures' websites.

Eukaryotic cell lines

Policy information about cell lines and Sex and Gender in Research

Cell line source(s)

Bone marrow stromal cells (BMSCs) were extracted from rabbit bone marrow, and cultured by using the previously reported protocol (Z.L. Li et al., Carbohydr. Polym., 2021).

Authentication

The BMSCs were authenticated by testing differentiation to a chondrocyte phenotype with chondrogenisis induction medium using the established protocol (J. Liu et al., J. Mater. Chem. B, 2017).

Mycoplasma contamination

We confirm that all cell lines were negative for mycoplasma contamination.

Commonly misidentified lines
(See ICLAC register)

There was no commonly misidentified lines for our study.

Animals and other research organisms

Policy information about studies involving animals; ARRIVE guidelines recommended for reporting animal research, and Sex and Gender in Research

Laboratory animals

Nude mice (age about 28 days, around 20 g, male) were purchased from GemPharmatech Co., Ltd. (Nanjing, China) for ectopic chondrogenesis test. BALB/C mice (6 weeks, 16-20 g, male) were purchased from the Laboratory Animal Center of Sichuan University (Chengdu, China) for in vivo inflammatory response test. Adult male New Zealand white rabbits (2.5-3.0 kg, 2.5-3 months old) were purchased from Dossy Experimental Animals Co.,Ltd. (Chengdu, China) for cartilage regeneration test. All mice were housed in a specific pathogen-free environment and kept in a room with controlled temperature (~25 °C) and humidity under 12 h light/dark cycle. Animals were housed 1-2 per cage and maintained on a 12:12 light:dark cycle (lights on at 8 AM) with controlled room temperature (~25 °C) and humidity (50-80%); and given ad libitum access to laboratory chow and tap water throughout the study.

Wild animals

No wild animals were used in the study.

Reporting on sex	The literatures about the relation between estrogen and chondrocyte regeneration have shown that estrogen could decrease cartilage thickness by inhibition of chondrocyte proliferation and increased chondrocyte maturation (Talwar RM et al., J Oral Maxillofac Surg. 2006;64(4):600-609). Therefore, in order to avoid the influence of estrogen on the evaluation of cartilage regeneration, we only choose male rabbits as experiment animals in this study.
Field-collected samples	No field collected samples were used in the study.
Ethics oversight	All studies of in vivo inflammatory response test, ectopic chondrogenesis and cartilage regeneration were approved by the permission of the Sichuan University Ethics Committee (protocol number KS2020330) and were carried out according to the institutional guidelines.

Note that full information on the approval of the study protocol must also be provided in the manuscript.

Plants

Seed stocks	NA
Novel plant genotypes	NA
Authentication	NA
